# China’s environmental solutions

**DOI:** 10.1007/s00253-022-12340-z

**Published:** 2023-01-10

**Authors:** Rolf Schmid, Xin Xiong

**Affiliations:** 1 Bio4Business and University of Stuttgart, Jagdweg 3, 70569 Stuttgart, Germany; 2 NMI Natural and Medical Sciences Institute at the University of Tübingen, Markwiesenstr. 55, 72770 Reutlingen, Germany

**Keywords:** China’s emission controls, Decarbonization, Eco-restoration, Bioeconomy

## Abstract

**Abstract:**

China emits unproportionately high concentrations of CO_2_ and, due to rapid population growth and industrialization, suffers from air, water, and soil pollution. However, many of these challenges for sustainable growth are being vigorously addressed, and China aims at a CO_2_ emission peak by 2030 and carbon neutrality by 2060 (“dual carbon policy”). In addition, nation-wide programs attempt to achieve reforestation and ecological restoration. By 2025, core elements of a “bioeconomy” and a circular economy are expected to be ready. Many of these programs extend into China’s international “belt-and-road” initiative (BRI). In this article, we briefly describe the present achievements of China’s environmental solutions and the country’s visions for a “digital, eco-friendly civilization.”

**Key points:**

• *China’s steps towards environmental cleaning, eco-protection, and decarbonization.*

• *Steps towards a future bioeconomy.*

## Introduction

Since its opening up in 1979, China has seen a strong increase in population, urbanization, and industrial growth and within just 40 years has re-emerged as a leading world power — a position the nation held for many centuries before 1800 (Maréchal 2007). This growth is accompanied by impressive improvements in infrastructure such as highways and rails, a large increase in digital information resources, and significant spending on science and technology. Table 1 provides some data on the rapid timeline of these developments and China’s global share in 2020.

**Table 1:** Timeline of China’s recent growth (data are from the World Bank, the National Bureau of Statistics of China, or other official Chinese sources)

	1980	2000	2020	% global share (2020)
Population (million)	981	1263	1402	19
Urbanization (%)	19	36	61	20
GDP (B US$)	191	1215	14,700	17
Foreign currency reserves (B US$)	1.6 (1978)	166	3217	
Cement production (M t)	80	597	2395	57
Steel production (M t)	37	129	1064	58
Primary energy consumption (M t of standard coal)	24	1386	4073	23
energy consumption (M t of standard coal)/share of coal	603/72%	1971/70%	4983/57%	26
Infrastructure				
Private cars (M units)	0.2	6.3	280	~ 19
Railroad tracks (T km)	50	69	146	
Highways (T km)	0	16	161	
5G base stations (M units)			1.43	60
Satellites in orbit			550	7
R&D expenditure (% of GDP)	0.7%	1	2.4	
R&D expenditure (B CN¥)		89.6	2439	
R&D full-time equivalents (M persons)	0.67	0.92	5.24	
Patents granted	107	85,473	3,520,901	5.1% of global PCTs

Similar to the environmental damages in Western nations and Japan during industrialization, China has also greatly suffered from environmental pollution during its rapid rise (Gardner 2018). Air pollution was a particular hallmark as recently reviewed in a global context (Fowler et al. 2020). In addition, surface and groundwater, seawater, coasts, and soil were polluted, and forests and biodiversity were lost. An early Environmental Protection Law from 1979 was outpowered by growth, in spite of repeated updates. Following alarm signs such as the Beijing smog event of 2013, much stricter regulations and controls were enacted. From 2013 onward, environmental protection has become a priority policy issue, and in the wake of global initiatives to halt climate change, China follows her own programs such as the “Double Carbon Policy” of 2020 and the “Beautiful China Initiative” of 2018, with the aspiration to extend her “digital civilization” along the “belt-and-road initiative” (BRI). In this mini-review, we will discuss China’s environmental solutions under the aspects of pollution control, eco-restoration, and decarbonization.

## Solutions to cleaner air

### Challenges 

With China’s rapid industrialization since 1980, air pollution became increasingly unbearable in many regions. During January 2013, air quality in Beijing became excessively bad, and the maximum hourly concentration of fine particulate matter PM_2.5_ went up to 680 μgm^−3^ (Wang et al. 2014).As the “2013 Report on the State of the Environment in China” (MEE 2014) noticed, only 3 out of 74 cities met the WHO guideline value of an annual exposure of < 35 μgm^−3^, indicating that it had become an absolute need to set standards and regulate fine particulate matter and other air pollutant concentrations. Sources for PM have natural origins such as sea salt, desert dust, and forest and grassland fires, augmented by anthropogenic emissions from power plants, industrial processes, car exhausts, and the burning of crop residue in the field and residential heating. As China is under the influence of the Asian monsoon, PM_2.5_ concentrations of cities are lower in summer and high in winter, a challenge for populous and industrialized northern China (Rohde and Muller 2015).

### Stricter regulations

In September 2013, the State Council issued an “Air Pollution Prevention and Control Action Plan during 2013–2017, followed by a “Blue Sky Action Plan” in 2018. Stringent clean air rules were implemented. Small and highly polluting factories and outdated industrial capacities were phased out. Natural gas for cooking and heating was promoted in the residential sector, and emission standards were raised for industry, the residential sector, and vehicles (Zhang et al. 2019). A key rationale for these actions was an emission inventory for China (MEIC) covering the emission of ten air pollutants and greenhouse gases between 1990 and 2015 (SO_2_, NO_2_, CO, non-methane volatile organic compounds (NMVOCs), ammonia, PM_10_, PM_2.5_, black carbon BC, organic carbon OC and CO_2_) (Li et al. 2017). The inventory came to the conclusion that industry, households, vehicles, and power plants made the strongest contribution to total emissions (Table 2).

**Table 2:** Relative contributions to China’s total emissions in 2010 (recalculated from Li et al. 2017)

Emission source	Total contribution	Dominant pollutant	Remarks
Industry	43%	NO_x_, SO_2_, PM_2.5_, CO, NMVOCs	Cement, iron, and steel plants and industrial boilers are major contributors of SO_2_, NO_x_, and PM_10_
Residential sector	35%	PM_2.5_, BC, OC and NMVOCs	Lack of data, but coal, oil, and gas and biomass for heating are important contributors
Transportation	11%	PM and CO	Tailpipe exhausts and evaporative emissions
Power plants	8%	NO_x_, SO_2_, PM_2.5_	Emission depends on type of coal (bituminous or anthracite) and type of boiler
Agriculture	4%	NH_3_, SO_2_, NO_x_, PM_2.5_	China largest emitter of atmospheric ammonia from fertilizers

The power of more stringent regulations was tested at the Beijing Asia–Pacific Economic Cooperation (APEC) meeting on November 10–12, 2014 (Zhao 2014). Attendants of this meeting in fact enjoyed a blue sky, dubbed “APEC blue,” due to the following measures:

-  ~ 10,000 factories in the Beijing region were closed, and an additional 39,000 ran on reduced operation.
- Twenty-four factories in Shanxi Province and 881 construction sites in Hebei province were closed, and another 1564 were under monitoring to be shut down, if necessary.
- State-owned enterprises, government offices, and schools were on a 6-day mandatory holiday.
- Over 60,000 industrial plants and 123,000 construction sites and petrol stations were closely inspected by over 400,000 cadres.
- From Nov. 7th until Nov 15th, heating systems from 12,255 coal-fired boilers were shut down.
- Only natural gas, no wood-based heating or cooking was allowed in a wide radius.
- 11.7 M automobiles were kept from the road, banning cars with even- or odd-numbered license plate every second day.
- Near the APEC site, 524 000 trees were planted.

Over the following years, additional measures for pollutant control were initiated. SO_2_, NOx, and PM_2.5_ emissions were lowered by modernization of power plants and industrial boilers, and residential heating by coal was reduced. Emissions from cars were another target. It became national policy to phase out older vehicles and tighten emission standards (Cheng et al. 2019). Fuel efficiency standards were based on weight and on corporate average fuel consumption (CAFC) limits for each manufacturer’s new vehicle fleet as a whole. The standards for 2020 and 2025 are 5 L and 4 L per 100 km, respectively. By April 2020, 16 regions in China representing 70% of the national market had all implemented China 6 (equivalent to EU 6). In 2020, the number of motor vehicles in China had reached ~ 370 M. Among these, personal cars were about 280 M or ~ 75%. About 14 M or 5% were trucks, 8 M among them heavy trucks of 8 tons or more. As of March 2022, a fleet of nearly 9 M battery and plug-in EV cars was the largest worldwide, and plans call for a share of electric vehicles and hydrogen fuel cell vehicles of 20% by 2025 and 50% by 2030 (He et al. 2020).

### Monitoring

Since 2012, air pollutants including PM_2.5_ are monitored by a Real-Time Air Quality Network, and data are provided to the public (https://www.aqistudy.cn/). In September 2022, this network comprised over 1499 stations in 367 cities throughout China (https://www.zq12369.com/datasource.php). A second sensor network of Sailhero Inc. includes 13,354 sites in 170 cities of 21 provinces. It monitors air pollutants, such as PM_2.5_, PM_10_, nitrogen oxides (NO_x_), carbon monoxide (CO), sulfur dioxide (SO_2_), ozone (O_3_), and total volatile organic compounds (TVOCs). Results can be retrieved online from a public website (https://www.aqistudy.cn) and are shown in the form of an air quality index (AQI) by a 6-color alert system.

### Trends

**Fig. 1:**
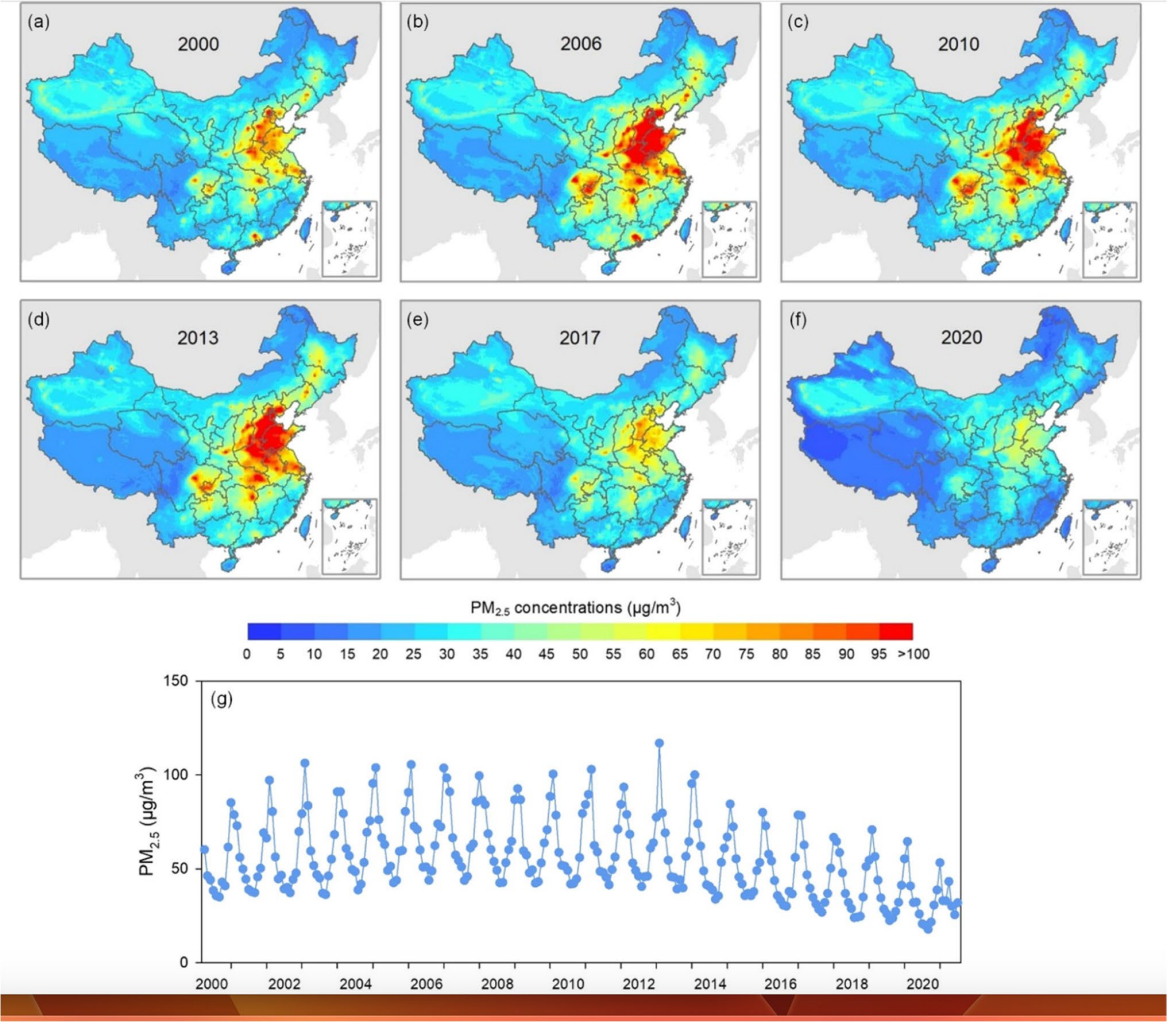
Development of annual mean PM_2.5_ concentrations over China from 2000 to 2020 according to the TAP database (Source: Geng et al. 2021)

Since 2001, the Ministry of Environment and Ecology MEE publishes annual reports on China’s environmental situation in English (MEE 2022). Concerning air pollution, the focus is on PM_2.5_, PM_10_, O_3_, SO_2_, NO_x_, and CO. In 2020, the air quality of 202 out of 337 cities reached the national standard. This is corroborated by a recent study based on the TAP database (http://tapdata.org.cn/), a comprehensive program on tracking air pollution in China. As shown in Fig. 1, in terms of PM_2.5_, air pollution has much improved over the past two decades (Geng et al. 2021).

## Solutions to clean water supply

### Challenges

China’s freshwater resources are not enough for the country’s huge population, and the annual per capita availability of fresh water is just about 2000 m^3^, one third of the global average (World Bank 2018). According to a review of the Ministry of Water Resources, total water supply in 2021 was 592 B m^3^, of which 83% came from surface waters. Seventy-nine percent of these supplies were consumed by primary industries, mostly agriculture and energy, and only 15% was available for households (MWR 2022). These are the challenges:

- Populous and industrialized Northern China receives only 20% of the country’s rainfall and snow melt, compared to 80% of southern China.
- Water supply is unevenly distributed over the year, and in most regions, precipitation in 4 consecutive months accounts for 60–80% of the annual total.
- Agriculture consumes about 2/3 of all water resources (rice cultivation requires plenty of water) and coal processing another 20%, leaving little water for the urban population.
- Runoffs from intense farming and pollution from industry and ships have resulted in poor quality of surface water.
- Sewage treatment has made much progress in urban China, but China’s countryside is still underserved.
- North China is rich in groundwater reserves, but over-exploitation and pollution of groundwater, e.g., by unregulated discharge of waste water, have led to a drop of groundwater level, the drying of aquifers, and the depletion of water sources.
- Along China’s coastline of ~ 14,000 km, sewage discharges, algal blooms, plastics, and microplastics are major issues of concern (Wang et al. 2021). According to a 2021 monitoring program of the MEE at 458 sources of emissions (from 1359 sources), 7.3 Bt of sewage was discharged into the oceans, with 141,841 t of COD and 583 t of petroleum. There was an average of 3.6 kg per 1000 m^2^ of floating waste, of which 92.9% was plastic (Baynes 2019). Some of the sources emitted mercury, hexavalent chromium, lead, and cadmium.
- “Green tides” besiege the coast of Shandong Province every summer. At the origin are *Ulva prolifera* algae whose bloom is caused by seaweed farming off the coast. Known as zicai 紫菜in Chinese and nori 海苔in Japanese, this edible seaweed is an important part of East Asian diets. *U. prolifera* is grown on rafts floating in shallow coastal waters, and after harvest, rafts are left at sea. The remaining algae join together to form huge floating masses which winds and currents push to the north towards the Shandong coast (Wang et al. 2015). Inactivation of the overwintering banks of *U. prolifera* algae on the cultivation ropes by a short pulse of chlorine dioxide, an environment-friendly disinfectant, made the green algae gradually disappear within 2 weeks after the treatment (Gong et al. 2021).

### South-to-north water diversion

The South-to-North Water Diversion Project is the largest of its kind ever undertaken. The project involves drawing water from southern rivers and supplying it to the dry north (Office of the South-to-North Water Diversion 2016). This massive scheme has already taken 50 years from conception and is expected to take almost as long to construct. Planned for completion in 2050, it will eventually divert 44.8 B m^3^ of water annually to the population centers of the drier north. When finished, the work will link China’s four main rivers — the Yangtze, Yellow River, Huai River, and Hai River — and requires the construction of three diversion routes, stretching south-to-north across the eastern, central, and western parts of the country. The complete project is expected to cost US$62 B — more than twice as much as the country’s Three Gorges Dam (Yu et al. 2017).

### Stricter regulations

Water quality in China is measured according to GB5749-2006 for drinking water and GB3838-2002 for surface water (Su et al. 2017). In 2015, the State Council passed the “Water Pollution Prevention and Control Action Plan” with the following targets and achievements by the end of 2019:

- By 2020, 70% or more of seven key rivers (Yangtze, Yellow River, Pearl River, Songhua River, Huai River, Hai River, and Liao River and their estuaries) should have a water quality at or above class III according to GB 5749–2006, and by 2030, this figure should exceed 75% (targets were achieved by the end of 2019).
- Each prefecture-level city should have centralized water treatment facilities, and there should be a maximum of 10% of black and odorous water bodies.
- By 2020, 95% of all urban centralized drinking water facilities should produce drinking water of at least class III quality (targets were achieved by the end of 2019).
- Water consumption should be reduced by 23% from 2015 levels (28% reduction was achieved).

In addition, urban sewage facilities were to be upgraded, the number of regional wastewater treatment plants to be increased, and the use of chemical fertilizers and insecticides had to be lowered, in order to reduce contamination from agricultural pollutants. By the end of 2019, the MEE reported that industrial clusters, communities, and farms had largely complied to these targets.

### Monitoring

China’s a network of hydrological observatory numbers some 121,000 observation points with 3154 stations, 14,286 points for surface water quality measurement, and 26,550 for the measurement of groundwater quality. Since 2019, the MEE operates a public website on surface water quality and discloses the top 30 cities with the best and the bottom 30 with the poorest water quality. A national groundwater observation network of 67,000 will increase to 76,000, soon covering 7.4 M km^2^ out of China’s 9.6 M km^2^, in particular areas with vulnerable ecology (Zheng 2022). Since 2019, a Beijing-based NGO, the Institute of Public and Environmental Affairs (IPE), publishes its own water quality standard, the “Blue City Water Quality Index (BCWQI)” (Shen 2019). It is based on a score of the local quality of surface water (50%), drinking water (30%), and groundwater (20%), using the standard water classification system (class I–V) (https://www.ipe.org.cn/MapWater/water.html?q=2).

### Satellite and robot survey

Water quality monitoring by satellite remote sensing remains challenging due to low signal-to-noise ratios (SNRs) and instrumental resolution limitations. Proximal remote sensing may be a more immediate solution (Sun et al. 2022). Along its coastal line, China now deploys unmanned robots “Jinghai “ to collect data and take videos. They conduct automatic topographical mapping, sea floor exploration, and environmental monitoring (https://jhai.shu.edu.cn/wrtjs/jh1h.htm).

### Sewage treatment, pipes, and sludge

As of September 2019, China had 5333 wastewater treatment plants (Xu et al. 2020), trickling filters in smaller communities and activated sludge plants such as the Gaobeidian Water Reclamation Plant in Beijing with a treatment capacity of 1 M m^3^/day. By the end of 2020, total length of drainage pipelines (sewers) was 803,000 km, with a distribution roughly corresponding to the regional GDP (Wang et al. 2021). There are still concerns that many of these plants do not clean at a desirable ratio (Zhang et al. 2021), and the sewer systems leading to these plants are still incomplete (Wang et al. 2021). Biological treatment of industrial wastewater is advancing (Mao et al. 2021). Sludge production in China in 2019 was 39 M tons or 28.1 kg/year per capita. More than half of the sludge was used for composting or sanitary landfills, only 27% was incinerated (Wei et al. 2020). The appropriate disposal of sludge generated during wastewater treatment remains an important task for the coming years.

### Trends 

**Fig. 2:**
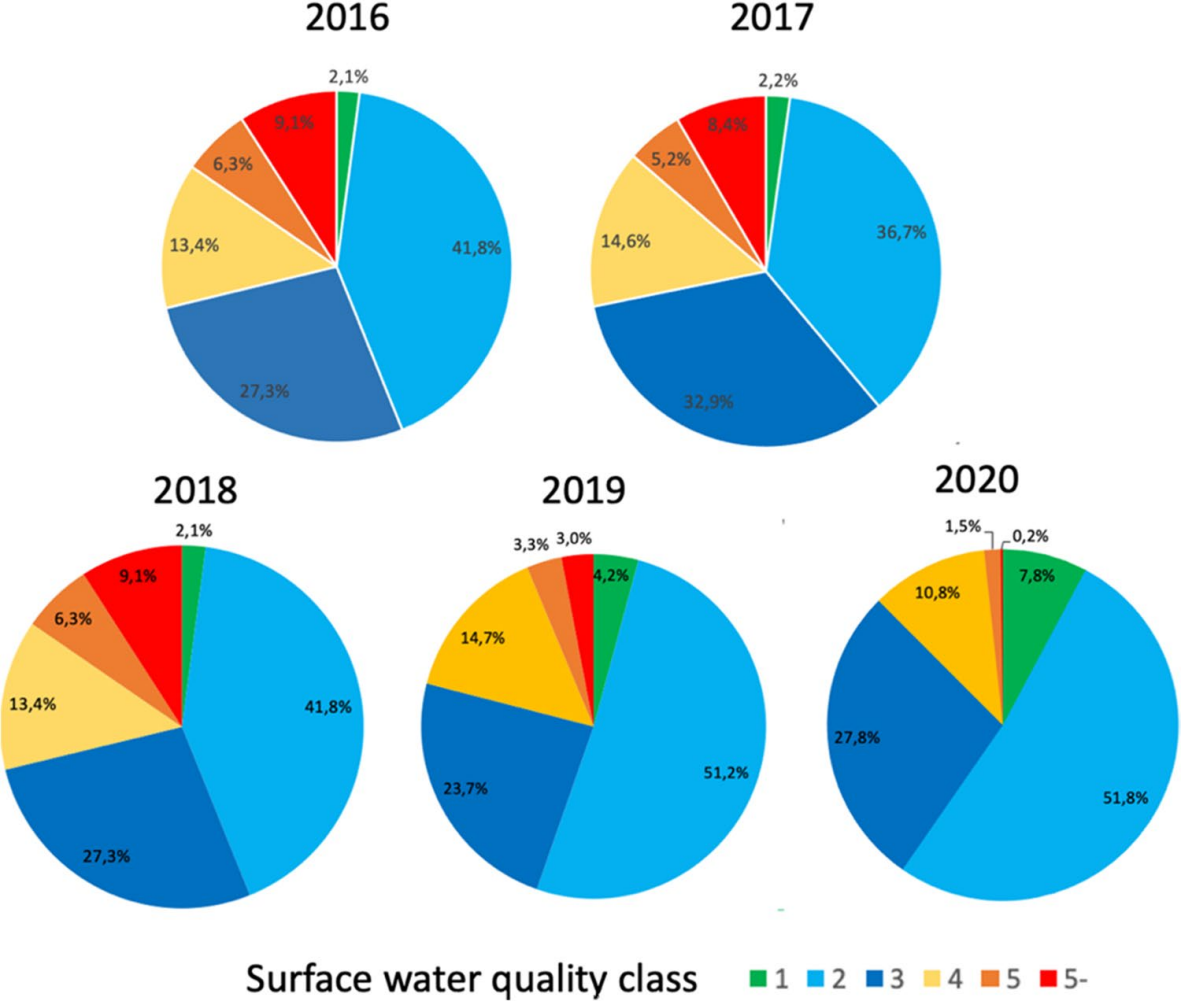
Water Quality improvement from 2016 to 2020 (Source: Ministry of Environment and Ecology, Water quality reports 2016, 2017, 2018, 2019, and 2020)

Based on data from the MEE, water quality has improved during the “135” period (Fig. 2).

Based on the BCWQI, water pollution levels had decreased by 2018. However, in a 2020 study of 744 publications on water quality research from 2007 to 2018 with data from a total of 680,265 measurements, the authors concluded that the overall quality of drinking water in China was still quite low, especially in rural and economically underdeveloped areas (Wu 2020). Microorganisms were the main factors affecting safety. There was also a high content of arsenic and fluorine in groundwater in some areas of China. The main factors affecting drinking water safety were (a) pollution of water source, (b) old water supply facilities, (c) aging pipe networks, and (d) inadequate treatment of drinking water. Records from 142 lakes and reservoirs showed that eutrophication is decreasing but contamination with heavy metals such as Cr and Cd or inorganic As are on the rise since 2012 (Huang et al. 2019), due to higher industrial production and energy generation based on coal. Antibiotics and antibiotic resistance genes could be detected in most surface waters and their sediments (Zhang et al. 2020).

## Soil and waste

### Introduction 

Many urban soils in China are heavily polluted due to rapid industrialization, coal firing, and increase of traffic. China’s urban population of > 700 M generates municipal solid waste (MSW) at a level of ~ 500,000 t/day. As the urban population is expected to become more affluent and rise still further, MSW is expected to increase to > 323 million Mt/year by 2030 (Kurniawan et al. 2022).

### Challenges

Uncontrolled dumping of shopping bags and mulching foils has led to “white pollution” in many places. In spite of prohibitions, this problem has not been solved, as controls are quite difficult (Zhao et al. 2020). Based on results from 100 sampling sites, polyaromatic hydrocarbon (PAH) pollution is strongest in China’s north and northwest, with an average of 900–1500 ng PAH/g soil (Zhang and Chen 2017), and also in coastal areas (Dai et al. 2022). Next to PAH, heavy metal pollution is severe in many places. Metals such as Cr, Cd, Hg, and As originate from mines, industry, coal firing, traffic, and other sources. It has been estimated that 25% of all agricultural soil in China contains excessively high Cd levels (Tang et al. 2016). From a review of 1203 publications between 2008 and 2018 on heavy metal pollution of soils in China, it was concluded that Hg and Cd were the most polluting heavy metals and that most polluted locations were in Hunan, Guangxi, Yunnan, and Guangdong Province (Hu et al. 2020). Antibiotics such as tetracyclines and quinolones have been widely detected in soil samples and are attributed to the use of manure as fertilizer and the reuse of domestic wastewater (Lyu et al. 2020).

Until recently, the principal procedure to deal with municipal solid waste was collection and dumping into landfills in the city outskirts. A catastrophic landslide event occurred in Shenzhen in 2015 because of overfilling of the landfill, injuring 17 people and killing at least 73 (Yang et al. 2017).

Waste cooking oil (“gutter oil”) is often released into the environment and pollutes soil and water (Zhang et al. 2012). Recycling of waste oil had increased from 65% in 2013 to 75% in 2018, and the first companies began to produce biodiesel from recovered waste cooking oil (Wang et al. 2020a).

## Solutions to soil remediation and garbage reduction

Remediation of heavy metal–polluted agricultural soils has been studied using soil amendments such as biochar or clay, phytoremedition using metal-accumulating plants, and foliar sprays containing Zn or Si (Qin et al. 2021). Soil can also be remediated by chemical and thermal processes. As soil remediation is both technically difficult and costly, heavily polluted or high-risk areas such as mining neighborhoods are high on the agenda (Hou 2021).

For household garbage reduction, the State Council had set a target to reach 35% by 2020 through sorting and recycling. From mid-2019, Shanghai has put into effect compulsory regulations, and other cities have followed this lead. In Shanghai, Alibaba’s widely used smartphone app Alipay offers a “Garbage classification guide” that has indexed over 4000 different types of waste. Nanjing has introduced “trash banks.” The residents first perform face recognition and then sort out the trash. The system returns points to the user’s account based on the number of recyclables contained in the garbage. Eventually points can be turned into cash. In Chongqing, the user identifies himself by facial recognition and puts the “wisdom container” into operation, which sorts the waste using artificial intelligence (AI) technology or printed QR codes on recyclable plastic bottles. The production of hydrogen-rich syngas from MSW by pyrolysis/gasification is a promising waste-to-energy pathway for realizing a circular bioeconomy (Lee 2022).

### Trends

According to a 2017 poll, more than 70% of the interviewed persons found that personal and consumers’ behavior are relevant for a clean environment. However, a more recent survey showed that the level of public engagement in government projects still falls short of expectations (Yin et al. 2021).

## Eco-restoration

### Introduction

Out of China’s land mass of ~ 9.6 M km^2^, only 15% is arable land. Four percent is used for constructions, including 17 cities with a population of > 10 M and over 90 cities with more than 1 M inhabitants. Roughly 1/4th each of China’s land are either grass plots, forests, or deserts (data are derived from satellite mapping and thus provide some overlaps). Extensive afforestation is used to mitigate dust pollution and claim new agricultural land. China’s environmental history, which has been carefully described (Marks 2017), knows repeated episodes of deforestation, the most recent during China’s industrial rise, rapid population growth, and urbanization.

### Challenges

Extending over many climate zones, China has a rich biodiversity which accrues, for example, the largest number of bird species and gymnosperm varieties in the world. Neglected for long, ecological conservation has recently become a major point of concern. Since China became a member of the UN Convention on Biological Diversity in 1994 (Cartagena protocol), ever more and stricter rules and regulations for eco-environmental protection were implemented. In 2011, a “Biodiversity Strategic Plan” was established with strong commitments towards a sustainable ecology (Xu et al. 2012), recently supported by artificial intelligence (Hua 2022).

## Solutions to eco-restoration

### Deserts 

China’s 13 deserts cover about 27% of the country’s landmass. The Taklamakan desert in Xinjiang Autonomous Region extends over 330,000 km^2^, and the Gobi desert spans even 1,570,000 km^2^, extending into the Mongolian Republic. Deserts and dried up steppes are a continuing source of sandstorms and at the origin of dust pollution in Beijing and elsewhere. Afforestation programs attempt to mitigate dust pollution and to claim new agricultural land. During the 13th Five-Year Period (“135”), desertification prevention work was done on 110,000 km^2^ of land (Global Times 2021). At present, the deserts shrinks by an average of 2000 km^2^ per year.

### Forests

Between 1990 and 2015, China increased forest cover from 17% of its territory to 22.5%, an increase of 512,000 km^2^. With the “Grain for Green” program of 1999, the government initiated an ambitious program against flooding and soil erosion, and some 40,000 km^2^ of land was planted with trees every year. This largest ecological construction project in human history involved 124 M people in 25 provinces. According to the National Forestry and Grassland Administration NFGA, China planted 338,000 km^2^ of forests between 2013 and 2018 and has set a target to achieve forest cover over 30% of its land by 2050 (Rapid transition 2018). This will involve 100 B trees to be planted in a belt covering more than 1/10th of the country. There will be red lines for forests, grasslands, wetlands, and sand protection, and the plan calls for natural forests where logging for commercial purposes is completely prohibited. A 2019 report based on satellite data from NASA showed that the leaf area of vegetation in China came by 42% from forests and by 32% from cropland, the rest being pastures (Chen et al. 2019). Between 2001 and 2017, China, with only 6.6% of the global vegetated area, accounted for 25% of the net increase in global leaf area. The Chinese Academy of Sciences CAS has established a “China Ecosystem Research Network (CNEN), which provides long-term continuous observations of atmospheric, hydrological, vegetation, and land elements of oasis–desert ecosystems.

### Forest villages

In 1982, 21% of a Chinese population of 1 B were not living on farms but in 130 cities and 2968 towns. In 2021, the number of urban residents had grown to 60% of 1.4 B people living in more than 684 cities over 500,000 residents. As a counterbalance to this rapid urbanization, China has established a first batch of 7586 “national forest villages” for rural greening and revitalization. They are located near waterways, roads, and rails and supplied by a modern infrastructure including IT (Gong et al. 2022).

### Biodiversity

In October 2021, the State Council released a white paper on “China’s Biodiversity Conservation,” the first governmental document of this type (State council 2021) in the framework of the United Nations “Convention on Biological Diversity” COP15-meeting in Kunming, Yunnan Province, China, as the host and over 100 nations signed the “Kunming declaration” (Kunming Declaration 2020). The 2030 Agenda for Sustainable Development was reaffirmed in view of the Convention on Biological Diversity and the 2050 Vision for Biodiversity. The Chinese term for these goals has become “ecological civilization,” and China has registered 34 locations under UNESCO’s biosphere project.

### Medicinal plants

An important aspect of biodiversity protection is China’s long experience on “traditional Chinese medicine” (TCM), based on a unique knowledge of indigenous materials from plants, animals, or minerals and in their use for the treatment of ailments and diseases. TCM products make up one third of the Chinese drug market. Over 2000 Chinese manufacturers hold government licenses for their production and over 200,000 for trading and sales, mostly in TCM pharmacies. As a consequence, the government protects medicinal plant diversity. In 2016, the State Council adopted a plan for the “Protection and Development of Chinese Phytotherapy (2015–2020).” It provides a whole bundle of measures to train farmers involved in their cultivation. In 2021, after 13 years of work, a book series in 13 volumes on “Chinese Medicinal Plants” included about 12,000 entries (Tian 2021); nearly 7300 of them came from the Sichuan province of Central China, dubbed “China’s pharmacy.”

### Botanical gardens and national parks

China counts about 200 botanical gardens and arboreta (Hu 2022). A famous example is Xishuangbanna Tropical Botanical Garden in Kunming, founded in 1997. In 2003, the Chinese Academy of Sciences (CAS) built a large botanical garden in Xi’an. In April 2022, China’s National Botanical Garden was opened in Beijing on 600 ha land. It covers 80% of the families and 50% of the genera of Chinese plant species. In July 2022, a 300 ha South China Botanical Garden in Guangdong followed suit. GenBanks of rare and endangered plants were built within the Qinling Mountain, Wuhan, Xishuangbanna, and Beijing. Two hundred fifty wildlife breeding centers were established throughout the country. As a result, the wild population of the giant panda remained at more than 1000, and the red ibis population increased from seven to > 500 birds. The population of artificially bred Chinese alligators is now nearly 10,000. Sightings of tigers have been reported in the northeastern, eastern, and southern parts of China. Since the 12th Five-Year Period (“125”), the CAS has accelerated various programs on China’s biological resources, integrating botanical gardens, specimen museums, resource banks, biodiversity monitoring networks, and experimental animal platforms to build a complete biological resource system. In December 2019, CAS published an online catalogue of the country’s biological resources (https://www.casbrc.org/en/index.html#/). It contains 7 million records from 40 CAS research institutes, including information on the Biodiversity Monitoring Network (Sino BON) and an overview in English. In 2017, the State Council ordered all provinces and regions to establish an “ecological red line” that will declare designated regions (National Parks) under mandatory and rigorous protection. The first 11 National Parks cover some 200,000 km^2^ and include Sanjiangyuan (the source of China’s three major rivers), giant panda habitats, and the Great Wall (Yang 2021a). Additionally, 474 national natural reserve regions were defined, where environments, animals, and biodiversity are highly protected. Surveys have changed from patrol workers to drones and IR cameras, e.g., for the registration of snow leopards in Sichuan Province, or gibbon monkeys and wild elephants in Yunnan Province.

### Resource centers

Food security has been of key importance throughout Chinese history. In the context of eco-restoration, a national seed breeding base is being completed on Hainan Island. Gene banks of rare and endangered plants were built within the Qinling Mountain, Wuhan, Xishuangbanna, and Beijing. China’s National Gene Bank was opened in 2016. It is located in Shenzhen, Guangdong Province, and serves as a biorepository for plant, animal, and microbial cells, as a bioinformatics data center and as a gene synthesis and genome editing platform (accessible at https://db.cngb.org). In 2017, another large biobank was opened in Shanghai’s Zhangjiang Science Park. It has a storage capacity for 10 million samples of tissue, cells, organs, blood samples, and other and stores 500,000 tumor tissue samples (accessible at https://www.shbiobank.com/category_199.html). The Chinese General Microbiological Culture Collection Center CGMCC founded in 1979 is located at the CAS Institute of Microbiology in Beijing (accessible at http://www.cgmcc.net/english/product.html). It holds some 40,000 microbial strains. The China Center for Industrial Culture Collection CICC, also located in Beijing, is affiliated with the National Research Institute of Food and Fermentation Industries (accessible at http://www.china-cicc.org). It holds a collection of ~ 10,000 microbial strains, most of them of industrial relevance.

## Solutions to climate change and decarbonization

### Introduction

China, with 19% of the global population, accounts for 30% of global greenhouse gas emissions — more than 10 G t/y of CO_2_. On a per capita basis, however, emissions are at about 6.8 t of CO_2_ per person — less than half of industrialized nations such as the USA, Australia, or Canada (Nogrady 2021).

### Challenges

A long-term inventory of greenhouse gases from 2015 showed that CO_2_ emissions came mainly from energy generation in industry and power plants, whereas CH_4_ emissions did essentially come from coal mining, enteric fermentation in ruminant animals, and rice cultivation. Emission from agricultural lands and adipic acid production, used to produce nylon 66, accounted for over 50% of total NO_x_ emissions.

China’s energy mix is based on coal. In 2020, China’s proven coal reserves were 143 B t or some 13% of global reserves. Most of the coal is used for power production in industry or in China’s 1100 power plants, by far the highest number in the world. For reasons of national energy security, construction of 45 new coal-based power plants was started in 2020, to replace smaller, dirtier, and less efficient units. Steel, cement, and heavy manufacturing is predominantly backed by coal power boilers, and China has announced to build 18 new blast furnaces to support its leading role in these industries. Chemical industry is also backed by coal, and syngas made from coal is used to substitute oil or biomass for the production of methanol, ethanol, and olefins.

China’s oil reserves are estimated at 3.6 B t (2020), which is substantial but far from enough to cover the demand. China imports some 70% of its oil supply from Russia, the Middle East, Angola, Brazil, and Venezuela. Natural gas resources are small by world standards.

Out of China’s 10 M t of CO_2_ emission in 2021, 39% came from electricity generation. They show a promising trend: CO_2_ emission per kWh was reduced to 558 g, 35% less if compared to 2005 (NEEC 2022). This trend is attributed to the prevention of line losses (BJX News 2022) and the increase of non-fossil and clean energy resources (nuclear) which accounted for about 25% of all energy sources for electricity generation.

**Fig. 3:**
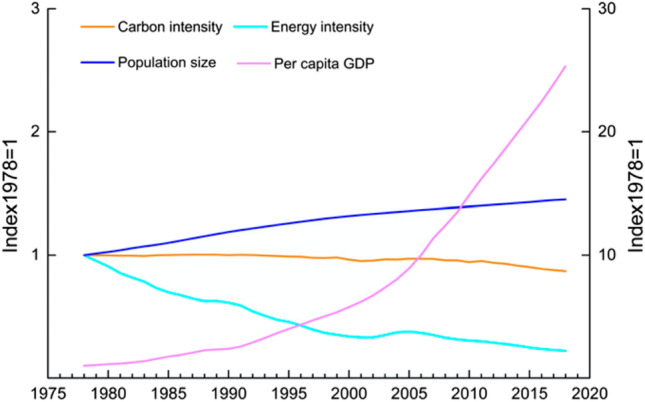
Timeline of the carbon and energy intensity of China’s economy (Source: Zheng et al. 2019)

China’s emissions between 1978 and 2018 increased from 1.37 to 9.64 Gt, with an average annual growth of 8.5% between 2001 and 2012. Since 2012, the annual increase of CO_2_ emissions has slowed down to about 1%, and both energy and carbon intensity (energy or CO_2_ emissions per unit GDP) are slowly declining (Zheng et al. 2019) (Fig. 3).

### Solutions to climate change

At the United Nations General Assembly in September 2020, Xí Jìnpíng proclaimed China’s “double carbon policy.” As a signee of the 2015 Paris Agreement on Climate Change, China will peak CO_2_ emissions by 2030 with a share of non-fossil energy of 20%, a 4.5 B m^3^ stock increase of forest and a carbon intensity reduced by 60–65% (CAT 2021). By 2060 at the latest, China should have become carbon–neutral. In the course of the 13th Five-Year Period (“135”), the share of coal in national energy consumption was already reduced to 58%. The current 14th Five-Year Period (“145”) calls to reduce, by 2025, carbon intensity by 18% and energy intensity by 13.5%. Key measures are investments in non-fossil energies, emission control, upgrading of manufacturing technologies, and development of carbon sinks. In February 2021, the State Council issued the “Guidance on Accelerating the Establishment of a Sound Economic System for Green, Low-Carbon and Circular Development.” In July 2021, the Ministry of Education followed up with an “Action Plan for Carbon Neutral Science and Technology Innovation in Higher Education Institutions.” Several research centers on decarbonization measures were established. Examples are the Research Center Carbon Neutrality Research at the CAS Institute of Atmospheric Science in Beijing and the Shandong Energy Institute in Qingdao.

### Reducing carbon footprints

**Fig. 4:**
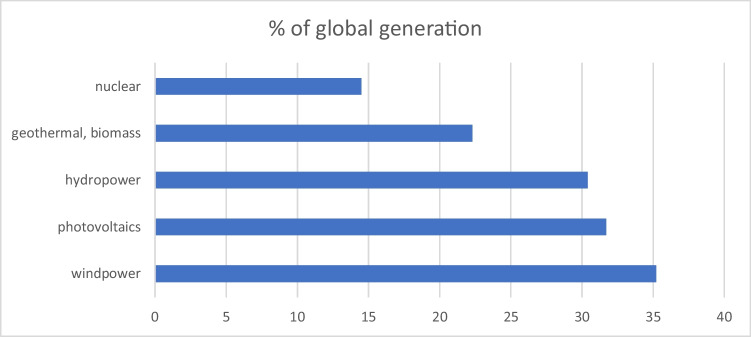
Share of China’s renewable and clean energies in global capacities (Data from BP 2022)

Among China’s conventional efforts are over 1000 modern coal-fired power plants with ultra-low emissions of nitrogen oxides, sulfur dioxide and dust (You 2021), a 10,000-ton demonstration project on steelmaking which uses “green hydrogen” (Lim and Pan 2022) instead of coal and near-zero-energy houses such as in Beijing’s Gaobeidian district (Shi and Liang 2021). The key issue, however, is that China has become a global leader in renewable and clean energy generation and generates already a larger share than its share in global population would suggest (Fig. 4).

*Hydropower* is China’s largest renewable energy source and has doubled since 2009 to 1.34 GWh in 2021 (http://www.gov.cn/xinwen/2022-01/29/content_5671076.htm). This is about 30% of the global hydropower capacity. The Three Gorges Dam on the upper Yangtze river with a capacity of 22.5 MW is the world’s largest power station but just one of several dozen more dams in operation or under construction. They serve not only for power generation, but also for flood control, water transportation, and water resource utilization. *Biomass* is a major energy source in China’s countryside. By 2019, almost 34 million farming households nation-wide used biogas digesters. Some 60 pilot towns and cities test the production of biogas from municipal solid waste. China is located both in the Himalaya and the circum-pacific geothermal belt (“ring of fire”). The total amount of *geothermal resources* is believed to account for about 8% of the world’s resources. About 80% of these resources are localized in the populated and industrialized East coast region, but the present installed capacity is only 28 MW (Wang et al. 2020). *Wind power* installed capacity in 2021 reached 328 GW and electricity production 655 TWh, the largest in the world (CEP 2022). It has been claimed that China could cover its whole energy demand by wind power (Song et al. 2021). The government has installed 6 windfarms with capacity above 1000 MW in Inner Mongolia, Gansu, Xinjiang, Hebei, and Jiangsu, the largest one near Jiuquan city with a nominal capacity of 20 MW. China’s Pacific coastline features shallow water depths of less than 60 m, offering low investment costs for wind farms (Song et al. 2021). By 2021, China had built 41 offshore windfarms of which 17 had a capacity over 100 MW (BJX 2021). China has the world’s largest installed capacity of *photovoltaics*. Its manufacturers dominate the world markets and could annually produce PV modules for over 50 GV electricity generation. In 2021, installed capacity was some 306 MW and contributed 327 TWh to nation-wide electricity generation (CEP 2022). Many large PV power plants are located in West China, an area with abundant sunshine and available land but sparsely populated. Heat can also be obtained from Concentrated Solar Power Plants (CSP) through a thermochemical reaction in molten salts or synthetic oil which drives a steam turbine generating electrical power. As of 2020, China operated one commercial CSP in the Gobi desert and explored 12 CSP elsewhere. China defines *nuclear power* as clean energy. As of early 2022, there were 54 nuclear power reactors in operation in China and 23 under construction. Installed capacity was 55 GV, contributing about 5% to China’s electricity consumption. Haiyang Nuclear Power Plant Unit 1, an AP-1000 reactor in Shandong Province, provides heating to the whole urban area of Haiyang with 200,000 residents, making Haiyang the first “zero-carbon” heating city in China (Zhao 2021). Pressurized water reactors are the mainstream design. Safety measures are a key issue, but final disposal of radioactive fuel waste — currently mainly via decay basins — has not yet been conclusively solved. China also operates a thorium molten salt reactor in the Gobi desert (Mallapaty 2021) and is a member of the international ITER project for the development of nuclear fusion reactors; the CAS Institute of Plasma Physics operates a comprehensive fusion research facility CRAFT on “Science Island” in Hefei, Anhui Province. China’s *hydrogen* production capacity is about 41 M t per year, but the present output is just 33 M t, mostly extracted from fossil fuels (H2CN 2021). According to the “Hydrogen Industry Development Plan to 2035” (NDRC 2022) of China’s National Energy Administration, hydrogen production using non-fossil energy sources should reach up to 200,000 t by 2025, and the build-up of a pertinent industrial chain is strongly supported by the central and local governments (Bai et al. 2021; Meng et al. 2021). Seventy percent of the production cost for hydrogen through water electrolysis are in electricity, and manufacturing cost must come down significantly, but companies such as Baofeng and Sinopec are already building PV-energized hydrogen production sites (XinHua News 2021), and Sinopec claims to have 1000 hydrogen gas stations ready by 2025, many of them along “hydrogen highways.”

CO_2_ capture, utilization, and storage (CCUS) is an emerging technology for low-carbon and high-efficiency development of fossil energy, which captures and purifies CO_2_ emitted during production and then reuses and stores it in a new production process. China Petrochemical Corporation has just started the first such project at Shengli oil field which will reduce CO_2_ emissions by 1 M t annually, equivalent to planting ~ 9 million trees (People’s Daily 2022).

### Carbon trading

China has implemented an emission trading system in 2020 and has announced a mandatory renewable energy certificate scheme for each of its provinces. China’s scheme is based on a cap-and-trade model, in which emitters — initially just coal- and gas-fired energy plants — are allocated a certain number of emissions allowances to a set limit, or cap, and then either trade or buy allowances if they remain below or exceed this limit. Each year, the cap is recalculated and reduced, providing an incentive to reduce emissions. As China expects its economy to grow by 4–5% per year, power consumption and emissions will further increase, but carbon trading prepares manufacturers to reduce their carbon emission per unit of economic output. As the integrity of reporting and monitoring emissions is essential, tighter reporting standards have been installed, independent agencies verify the data, and citizens are encouraged to report violations (Nogrady 2021). According to the Development and Reform Commission, a national and local carbon emissions accounting system will be established by 2023. It will feature a unified and normative carbon emissions accounting system with clear responsibilities. By 2025, China will be fully committed to the efforts for CO_2_ emission peaking out and carbon neutrality (Xinhua News 2022).

### Future trends: the “Beautiful China” Initiative

**Fig. 5:**
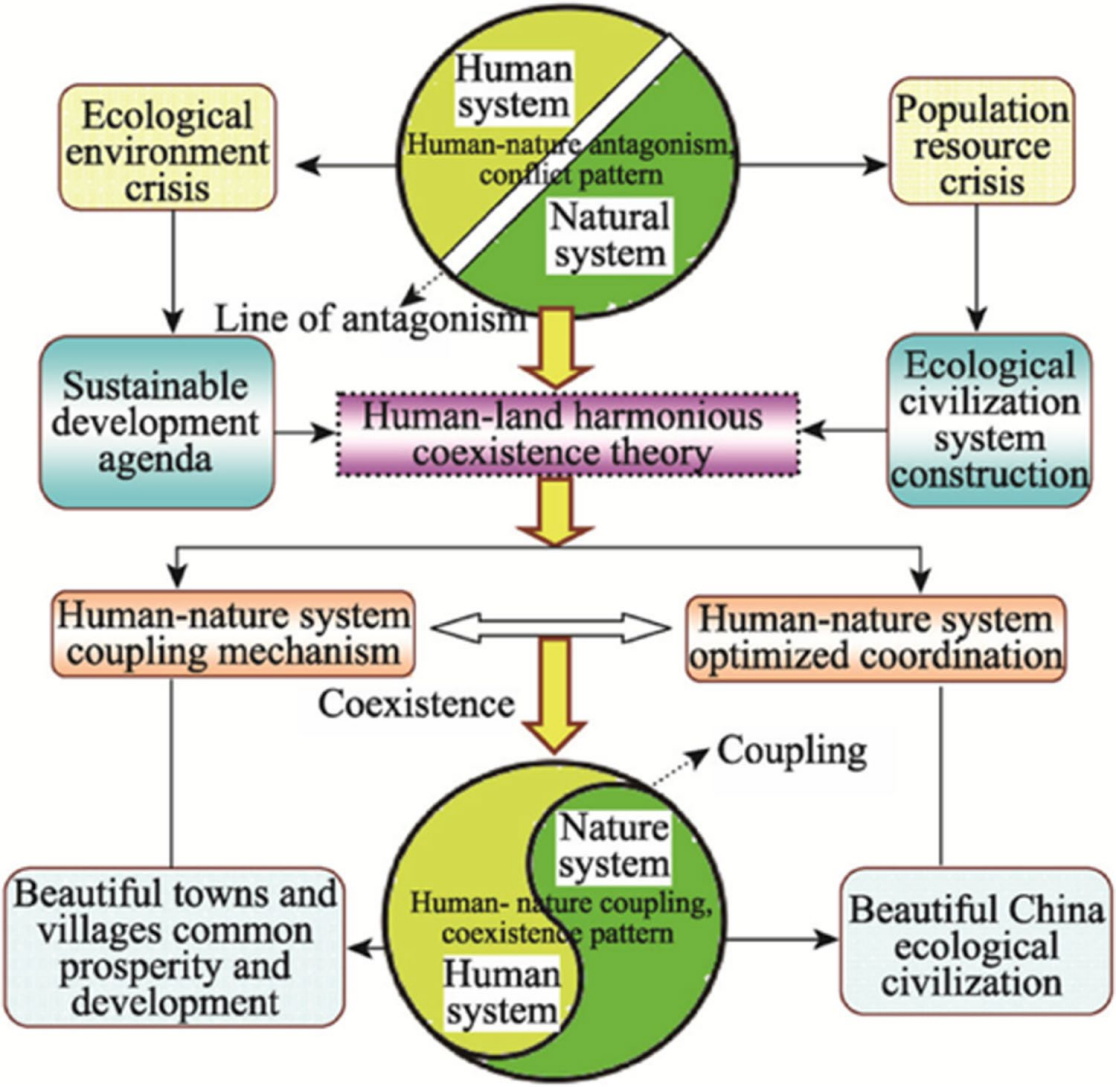
Scheme of the “Beautiful China Initiative” (Source: Fang et al. 2020)

Since the 12th and 13th 5-year planning periods, environmental protection has become a priority policy issue, summarized in the “Beautiful China Initiative” of 2018 (Fang et al. 2020). In this vision, shown in Fig. 5, a non-polluted, eco-friendly environment, as addressed in 4 out of 17 sustainable development goals of the United Nations, finds strong support by the Chinese government and is supported by various measures as described in this review (Taylor 2021). For the evaluation of progress, the CAS has established a “CAS Beauty Index.” It is based on the United Nation’s Human Development Index and evaluates 5 parameters, namely, (a) ecological environment, (b) green development, (c) social harmony, (d) system perfection, and (e) cultural heritage. As of 2018, an assessment of 341 prefecture-level cities in China showed that the CAS Beauty Index was still quite low, with an average score of 0.28 out of 1.0. Through pilot projects, performance indicators and dynamic monitoring and phased evaluations, a road map has been built which is expected to achieve an “ecological civilization” with harmonious coexistence of humans and nature in a “beautiful China” by 2035.

### 2035 vision: achieving socialist modernization basically

On its way to the 2049 centenary of foundation of the PR China, the Chinese Communist Party CPC targets 2035 as a midpoint milestone for comprehensive economic restructuring and technological breakthroughs which place China among the world’s leading countries, achieving an advanced standard of living, creating a green economy and an improved environment, and boosting per capita GDP while reducing the gap between urban and rural society. The present 14th Five-Year Period (2021–2025) is likely to see a shift from emphasis on economic growth to a focus on the sustainability of growth and the quality of life. It also foresees that domestic demand will be the main driver of China’s future economic growth. Specifically, the 14th Five-Year Period outlines renewed efforts to close the rural–urban income gap, promote global leadership in technological innovation, and increase the pace towards low-carbon development.

### Bioeconomy

In June 2022, the Chinese government announced its “Bioeconomy Development Plan for the Period of the 14th Five-Year Plan (2020–2025)” (Schmid and Xiong 2022). Priorities are summarized in Table 3.

**Table 3:** Priority topics in China’s bioeconomy program

Priority	Examples
Biomedical technologies	Personalized medicine, genetic screening, telemedicine, and remote diagnosis, more powerful traditional Chinese medicine TCM
Agriculture and food	Variety assurance and improvement, molecular plant, and animal breeding, “green,” circular, and environmentally friendly agriculture, health-promoting foods such as probiotics
Energy and materials from biomass	Biogas, ethanol from cellulose, algae biofuels, bio-kerosene, biodegradable mulch, and packaging materials
Safeguarding biological resources	National census for forests, grasslands, plant valuable materials including active ingredients for TCM, red list for biodiversity, satellite-based remote diagnostics and tracking, digital libraries, and resource banks
Leadership in key technologies	Synthetic biology, computational protein design, sequencing technologies
Biological security systems	National biosecurity and “global governance” in a changing world

The budget for basic research is being increased. It is flowing into new centers that conduct joint research with industry and into research clinics that conduct translational medicine. This package of measures is expected to create a large number of biotechnology companies with annual sales of over CN¥10 B (€1.5 B) with independent IP and first-in-class technologies. With international cooperation, the aim is to strengthen the still weak biotech sector and to develop an indigenous bioeconomy based on “Chinese wisdom and Chinese solutions” which will expand along the Silk Road Initiative. Particular funds will serve 4 bioeconomy pilot zones (a) the Beijing–Tianjin–Hebei metropolitan area, (b) the Yangtze River Delta (Shanghai–Jiangsu–Zhejiang), (c) the Greater Bay Area (Guangdong–Shenzhen–Hong Kong–Macau), and (d) the Shuangcheng Economic Circle (Chengdu and Chongqing). In July 2021, the NDRC published an update on China’s *circular economy* development during the 14th Five-Year period (Yang 2021b). By 2025, a resource recycling industry will be established worth 5 trillion yuan ($773 billion) which covers the entire society. Energy and water consumption will then be reduced by 13.5 and 16%, respectively, construction waste by 60%, and 86% of some 700 M t of crop straw annually will be utilized.

### China as a digital civilization

“Digital technology is fully integrated into all fields and the whole process of human economy, politics, culture, society, and ecological civilization construction with new ideas, new business forms, and new models, and has a broad and profound impact on human production and life” (Teller Report 2021). China has built up an impressive infrastructure to serve these goals. By mid-2022, China’s computing power was estimated at 27% of global capacity and leading in “smart computing” such as AI. Digital information systems included 1.7 million 5G base stations (70% of global share) which reached over 450 million 5G users among 1 billion Internet users. China had over 300 satellites in orbit, providing services of positioning, navigation and timing, global message communication, and remote sensing for weather, land survey, crop yields, and disaster monitoring. Gaofen satellites serve the “New Silk Road Space-Based Information Corridor” and so does the first SDGSAT satellite (for “sustainable development goals satellite”) launched in November 2021, to celebrate China’s 50th anniversary as a member of the United Nations. The Silk Road Initiative (belt-and-road initiative), initiated by Xi Jinping in 2015, comprises (1) the “Silk Road Economic Belt,” which roughly encompasses the historic Silk Road; (2) a “maritime Silk Road of the twenty-first century,” which extends via the ASEAN states along India and Ceylon to Africa and the Arab world; and (3) a “polar Silk Road” that seeks a maritime trade route along the Russian coast to Europe in the face of climate change. According to China’s Meteorological Administration, China has provided meteorological satellite data to 121 countries and regions, including 85 along the belt-and-road, and trained over 1400 people in their use (Xinhua News 2021). The “Earth Big Data Science” project of the Chinese Academy of Sciences includes a “digital twin” of the Earth, the “EarthLab” in Beijing. By these means, China strives to collect and compute data which are relevant to the UN’s sustainable development goals.

## Expectations up to 2035

Based on China’s current programs on environmental and ecological restoration, the coming period up to 2035, if undisturbed by major disruptions, might witness the following developments:

- Carbon caps, modernization of equipment, and continuing buildups in regenerative and clean energies will reduce the use of coal in industry and heat and electricity generation, translating into cleaner air over China.
- Coal will remain an important resource for the chemical industry, but processes will become more environmental friendly through the use of improved catalysts and clean or green energy such as hydrogen.
- Carbon emissions will peak around 2030, and carbon and energy intensity will decline.
- Lake and river pollution will decrease on a national scale due to stricter controls and more sewers and sewage treatment in smaller townships and the countryside. Marine pollution might remain a problem more difficult to solve.
- Soil remediation may see progress in urban space and industrial parks but remain recalcitrant in the countryside.
- Measures to stop desertification and to increase forests and cultivable land will progress.
- So will eco-restoration, through national parks, national reserves, and the use of high-tech support such as drones, satellite-based surveys and AI.
- In the framework of programs for a bioeconomy and a cyclic economy, industrial biotech will see a upward trend towards less wasteful processes, taking advantage on China’s huge resources of waste products such as straw or food waste.
- Science and technology will continue to play a leading role towards the development of a “digital, eco-friendly civilization” and science education of China’s citizens will play a pivotal role.
- Finally, China will continue to export environmental technologies to less industrialized countries, often through the belt-and-road initiative (BRI), and by these means will make an important contribution to the technical empowerment of hitherto less developed nations (Table 4).

**Table 4:** List of abbreviations

B	Billion
BRI	Belt-and-road initiative
CAS	Chinese Academy of Sciences
CN¥	Chinese Yuan
GDP	Gross domestic product
M	Million
MEE	Ministry of Environment and Ecology
PAH	Polyaromatic hydrocarbon
R&D	Research and development
S&T	Science and technology
T	Trillion
TCM	Traditional Chinese medicine
US$	US dollars
WHO	World Health Organization

## Data Availability

The data that support the findings of this study are available from the corresponding author, [RS], upon reasonable request.

## Declarations

### Disclaimer

The figures and information used in this article, unless indicated by citations, are taken from Internet sites of the World Bank, the Chinese Statistical Office, the relevant Chinese ministries and authorities, and the Chinese press. They are mostly in Chinese language and can be made available upon request. An extended version of this review is due to appear as a book in early 2023. Current information can also be found under www.window-to-china.de.
